# Poisonous Chloroform

**Published:** 1852-10

**Authors:** 


POISONOUS CHLOROFORM.

From the subjoined article, which we copy from the *Boston Medical and Surgical Journal* of the 8th of September, it will be seen that Dr. Jackson thinks he has discovered the cause of the many accidents which have resulted from the inhalation of Chloroform. Every philanthropist will rejoice if the conjecture of Dr. Jackson should prove to be well-founded. It cannot be disguised that there is danger in the use of this agent as commonly administered. The numerous deaths that have followed its administration have deterred many physicians from its use altogether, while they have rendered all apprehensive of its consequences. He will be a true benefactor of his race who shall disarm this invaluable remedy of its terrors. Dr. Jackson's communication is as follows:

The numerous deaths which have recently taken place from the inhalation of chloroform, seem to require that I should state what I know upon this subject, without waiting for more extended researches which I have now in progress; for a word in time may save human life, and I shall therefore present my views, even though some may think that I ought to wait until my work is completed to its full extent before publication. I have formerly been charged with dilatoriness in presenting my discoveries to the public, and wish to avoid a repetition of this accusation, even though my work, in its present state, is not so complete as would be required for scientific purposes.

I have long had a strong suspicion that the very sudden deaths resulting from the inhalation of chloroform, must have been produced by the presence of some poisonous compound of amyle, the hypothetical radical of Fusel oil, or the oil of whisky; and I began a series of researches upon this subject several years ago, but was called off from my work by unexpected persecutions. This work I have resumed, and I will now state what facts and inductions I am able to lay before the public:

1st. When chloroform, and the alcoholic solution of it called chloric ether, was made from *pure* alcohol diluted with water, no fatal accidents took place from its judicious administration.

2d. When chloroform was made, as it now frequently is, from common corn, rye, and potato whisky, deaths began to occur, even when the utmost care was taken in its administration.

3d. In the Chelsea case, where this kind of chloroform was probably contained in the alcoholic solution incorrectly called chloric ether, death took place in a very sudden manner, and the post-mortem appearances of the subject indicated the usual effects of poisoning by chloroform.

From these data, it might justly be inferred that some poisonous matter exists in the cheap chloroform of commerce, and I suspected that it arose from the Fusel oil which exists in whisky. This opinion, at my suggestion, was published by two of my friends, to put the public on their guard, and those gentlemen urgently advised that physicians and surgeons should return to the use of pure sulphuric ether, (oxide of ethyle,) as originally prescribed by me,

It is well known that I have always preferred my original anæsthetic agent to all the substitutes that have been proposed since; but still I have always been willing to give the proposed substitutes a fair trial, and did try them all, first upon myself, and then upon such of my pupils as felt willing to allow the experiment to be made upon them. I also, in a measure, compromised with that powerful anæsthetic agent chloroform, by mixing small proportions of it, about one-fourth or fifth part, with sulphuric ether, so as to concentrate the anæsthetic agent into a smaller bulk, and I have extensively used this preparation in the production of anæsthesia, and without producing any dangerous or even unpleasant symptoms in any case, but I always took care to ascertain that the chloroform used by me was pure.

Having, during the last month, succeeded in procuring some very pure Fusel oil, (of whisky,) I undertook the researches which have resulted in the conviction that it is this amyle compound that produces the poisonous matter of certain kinds of chloroform. When mixed with hyperchlorits of lime (bleaching powder) and water, in the same way as we prepare alcohol for the production and distillation of chloroform, I found that the mixture in the retort, after agitation and standing some time, became warm, indicating that a reaction was taking place between the Fusel oil and the hyperchlorite of lime.

After some hours the retort was placed in a water-bath, and distillation was effected, the volatilized liquid being condensed by one of Liebig's condensers. A clear colorless liquid came over, which was at once recognized as having the peculiar *odor of bad chloroform.* It is, perhaps, a *ter chloride of amyle,* but has not yet been submitted to analysis. It is so powerful that merely smelling of it makes one dizzy, and working over it made me so sick that I was obliged to go out of doors for fresh air several times during my operations on it. In order to make sure that the Fusel oil was all decomposed, I again mixed the product of the distillation above mentioned with a new lot of bleaching powder, and water; and after three hours, with frequent agitation, it was again distilled, and gave what I regard as the pure unmixed poison. This I am now to test on such animals as have proved good ether subjects, and shall make report of my results in this Journal.

If my views are correct, it follows:

1st. That all chloroform intended for inhalation as an anæsthetic agent should be prepared from pure rectified alcohol, to be diluted with water when used for distillation, from hyperchlorite of lime.

2d. That no druggist should sell for anæsthetic uses any chloroform which is not known to have been properly prepared as above suggested.

3d. That the mixture of chloroform and alcohol, commercially known under the name of strong chloric ether, must be made with the same precautions as chloroform.

There is less danger of the existence of Fusel oil in sulphuric ether, which is always made of strong rectified alcohol.

There is more danger of the existence of sulphurous acid in this liquid, and that is a dangerous poison, but it is one readily detected; and persons will object to inhaling ether containing it, on account of its well-known disagreeable odor of burning sulphur.

